# Supplementary material for: Using Wastewater Surveillance to Monitor Gastrointestinal Pathogen Infections in the State of Oklahoma
Source: Microorganisms. 2023 Aug 30;11(9):2193. doi: 10.3390/microorganisms11092193 (PMC10536226; doi:10.3390/microorganisms11092193)
Supplement: Supplementary file 1 [file microorganisms-11-02193-s001.zip › microorganisms-2564294-supplementary.pdf]

**Supplemental Table S1:** Details for qPCR and RT-qPCR of target organisms.

| **Organism (reference)** | **Target (gene/genome)[a]** | **Primer/ probe** | **Sequence (5'-3')** | **Conc (nM)** | **Reaction conditions** |
|---|---|---|---|---|---|
| *Campylobacter* [22] | *hipO* NC_002163.1 | Forward | TGCACCAGTGACTATGAATAACGA | 800 | 95 °C for 2 min, 45 cycles (95 °C for 20 sec, 60 °C for 1 min) |
| | | Reverse | TCCAAAATCCTCACTTGCCATT | 800 | |
| | | Probe | /56-TAMN/TTGCAACCTCACTAG CAAAATCCACAGCT/3IAbRQSp/ | 200 | |
| *Salmonella* [24] | *invA* U43272 | Forward | ACAGTGCTCGTTTACGACCTGAAT | 250 | |
| | | Reverse | AGACGGCTGGTACTGATTATAAT | 250 | |
| | | Probe | /5HEX/CGACCCCATAAACACCAAT ATCGCC/3IABkFQ/ | 100 | |
| Norovirus G-II [23] | GII.4 X86557 (pos. 4998 - 5107) | Forward | ATGTTCAGRTGGATGAGRTTCTC | 500 | 50°C for 20 min, 95°C for 2 min, 45 cycles (95°C for 30 sec, 60°C for 30 sec) |
| | | Reverse | TCGACGCCATCTTCATTCACA | 900 | |
| | | Probe | /56-FAM/AGCACGTGG/ZEN/GAGG GCGATCG/3IABkFQ/ | 250 | |

[a] gene or genome name, Genbank accession, and nucleotide position if not entire gene
